# Supplementary material for: MetaQA: Enhancing human-centered data search using Generative Pre-trained Transformer (GPT) language model and artificial intelligence
Source: PLoS One. 2023 Nov 13;18(11):e0293034. doi: 10.1371/journal.pone.0293034 (PMC10642800; doi:10.1371/journal.pone.0293034)
Supplement: Attachment — Submitted filename: Response to Reviewers.docx [file pone.0293034.s002.docx]

Dear Reviewers,

We extend our sincere gratitude for your meticulous feedback and constructive suggestions on our manuscript, "MetaQA: Enhancing Human-Centered Data Search using Generative Pre-trained Transformer (GPT) Language Model and Artificial Intelligence". We have carefully considered your insightful comments and have implemented appropriate revisions to address your concerns. Please find our point-by-point responses to your feedback below.

**Reviewer #1:**

- **Pertaining to the type of sentences that better suit the problem and the influence of input sentence quality on the output:**

*We appreciate your thoughtful inquiry. In the context of a question-answering task employing a masked language model, the input sentence should ideally be a query that directly pertains to the data represented in the table. It should be articulated clearly and concisely to ensure the model's accurate understanding of the question and subsequently, a precise response. Ambiguities or excessive complexities may detract from the precision of the answer.*

*Given that the model is essentially a statistical pattern matcher trained on large datasets, it can only provide answers based on patterns it has learned during training. Thus, if the input query does not align closely with these patterns, the model may encounter difficulties in generating high-quality responses.*

*Consequently, the quality of the input sentence significantly influences the quality of the output as it primes the model for pattern matching during the question-answering process.*

*We have revised the original paragraph to clarify this point; please refer to the highlighted section in Lines 215 - 227.*

- **Regarding the sentence about the training architecture that lacked clarity:**

*We apologize for the confusion caused and have modified the sentence to ensure better clarity. The revised sentence now states: "We adhered to the table pre-training via execution [15] training architecture, which involves applying tabular question-answering downstream tasks with an end-to-end workflow. This begins with a BART base model pre-trained as a free-form language model to conduct the SQL synthetic. Subsequently, the pre-trained language model is fine-tuned using a multi-round tuning method with various datasets, including the Wikipedia Question Answering Dataset and the Metadata Question Answering Dataset."*

- **Regarding the low resolution of figures:**

*We have enhanced the resolution of all the figures, including Figure 1, to facilitate better readability.*

- **Concerning typos:**

*We have rectified the typos pointed out by you and also performed a comprehensive review of the manuscript to eliminate any remaining errors.*

**Reviewer #2:**

- **About the evaluation on a single dataset:**

*We acknowledge your concern regarding the robustness of our method. To this end, we have evaluated our model on an additional Table QA dataset. The results from this are included in the revised manuscript after Line 392. These results corroborate the effectiveness of our method on multiple datasets, thereby strengthening our claim of robustness.*

- **About the statistical strength of the experiments:**

*We concur that the inclusion of statistical values is fundamental to establishing the validity of our findings. We have now provided the average and standard deviation from 10 independent experiments with different random seeds in the revised manuscript. We have also specified the values of the random seeds in Lines 339 - 340.*

- **Regarding data and code availability:**

*We understand the importance of data and code accessibility for reproducibility. In line with the PLOS Data policy, we have made the Metadata Question Answering dataset privately available for peer review via Dryad, an official data repository for research publications. Here is the link:* [*https://datadryad.org/stash/share/iBAzVXtzlu_-mBVXbKuAu66i8raXZZb1SYKtO84ReLE*](https://datadryad.org/stash/share/iBAzVXtzlu_-mBVXbKuAu66i8raXZZb1SYKtO84ReLE)

*Furthermore, we have updated the size of the dataset according to our funded project's increasing volume. However, as much of the code is used in our funder’s project, we may not be able to make the entire code publicly available immediately. We will continue to communicate with our funder and carry out Code Sanitization in all details of our Code and Data as the project and publication progress.*

- **On redundant content and minimal implementation details:**

*We have thoroughly revised the manuscript to remove any redundancies and have bolstered the depth of the implementation details to provide a more comprehensive understanding of our method. Specific revisions include:*

*In the abstract, we scrutinized the grammar and excised the superfluous description of the GCOOS interface. In Lines 1 - 9, we removed unnecessary information and streamlined our language to ensure readability and clarity.*

*In Lines 17 - 18, we eliminated the current search results introduction of the traditional method to avoid repetition.*

*In Lines 36 - 45, we simplified our discussion of the FAIR principle and reframed the introduction of the language model to more directly and effectively convey the central ideas.*

*Lines 81 - 89 saw the removal of redundant background information on the GCOOS platform. We believe this change allows the reader to focus more on the core substance of our research without being distracted by superfluous context.*

*In Lines 91 - 102, we overhauled the background of the traditional keyword-based search method to ensure that it more effectively complements and supports our method.*

*In Lines 116 - 125, we refined our description of relevant question-answering research to enhance the context and background against which our research is positioned.*

*Finally, in Lines 129 - 136, we reworked the discussion of the challenges of question-answering models to better highlight the issues our research seeks to address.*

*Additional modifications, indicated in the highlighted text within the manuscript, pertain to grammar corrections and typo fixes.*

*We hope that these modifications have improved the readability and clarity of our manuscript and look forward to your further feedback.*

- **Regarding the quality of figures and their placement:**

*We have enhanced the quality of the figures for improved readability and have integrated them within the manuscript to facilitate better comprehension.*

- **Concerning typos:**

*We have corrected the typos mentioned and conducted a thorough review of the manuscript to identify and rectify any remaining errors.*

We trust that our revisions have satisfactorily addressed your concerns. We greatly value your time and effort in reviewing our manuscript. We are confident that these modifications have considerably enhanced the quality of our work and we look forward to receiving further feedback.

Sincerely,

Diya
